# Community-based Health Planning and Services programme in Ghana: a systematic review

**DOI:** 10.3389/fpubh.2024.1337803

**Published:** 2024-03-05

**Authors:** Abena Boahemaa Adusei, Helen Bour, Hubert Amu, Augustine Afriyie

**Affiliations:** ^1^Department of Epidemiology, Noguchi Memorial Institute for Medical Research, University of Ghana, Accra, Ghana; ^2^Department of Community Health, Ensign Global College, Kpong, Ghana; ^3^Department of Population and Behavioral Sciences, University of Health and Allied Sciences, Ho, Ghana; ^4^Department of Medicine, Psychiatry, Obstetrics and Gynecology, Eastern Regional Hospital, Koforidua, Ghana

**Keywords:** Community-based Health Planning and Services, Universal Health Coverage, Primary Health Care, Community Health Management Committee, Community-Based Health Volunteer, Ghana Health Services, Ministry of Health

## Abstract

**Introduction:**

Ghana established Community-based Health Planning and Services (CHPS) as the primary point of contact for primary healthcare in 1999. CHPS has since emerged as the country’s primary strategy for providing close-to-client healthcare delivery, with numerous positive health outcomes recorded as a result of its implementation. There is, however currently a paucity of systematic reviews of the literature on CHPS. The purpose of this study was not only to investigate dominant trends and research themes in Community-based Health Planning and Services, but also to track the evolution of the CHPS intervention from its inception to the present.

**Method:**

We adopted a systematic review approach for selected articles that were searched on Google Scholar, PubMed, and Scopus databases. The study was conducted and guided by the Preferred Reporting Items for Systematic Reviews and Meta-Analyses (PRISMA) guideline. We then applied a reflexive thematic analysis approach in synthesizing the results.

**Results:**

The search resulted in 127 articles of which 59 were included in the final review. Twenty (20) papers targeted the national level, eighteen (18) for the regional level, sixteen (16) for the district level, two (2) for the sub-district level, and three (3) papers targeted the community. The years 2017 and 2019 were recorded to be the years with the highest number of publications on CHPS in Ghana.

**Conclusion:**

Community-based Health Planning and Services (CHPS) is an effective tool in addressing barriers and challenges to accessing quality and affordable health care causing significant effects on health. It provides close-to-client healthcare delivery in the community.

## Introduction

The Alma-Ata Declaration of 1978 represented Ghana’s third major national attempt to provide health care for all of its citizens. Prior to that, both the comprehensive colonial policy of Sir Gordon Guggisburg (in the 1920s) and the very aggressive Kwame Nkrumah policy (1960s) after independence could not provide health for all Ghanaians in a sustainable way (1–5). Despite the monumental significance of the Alma-Ata declaration, there is overwhelming research evidencing the major health disparities in the country and how the poorest have almost been neglected in the distribution of healthcare resources even at present (1–5). Some researchers attribute the failings in all these major national projects to the inability of the healthcare system to connect to the community members in ways that promote equity, social justice, and participation (6). In Ghana, Community-Based Health Planning and Services were designed specifically to fill this gap (7). Over 40 years after the Alma-Ata declaration, Universal Health Coverage (UHC) remains an illusion for many poor communities across the country (8). Despite all the reported successes of the CHPS process, there are wide disparities in the effectiveness and coverage of CHPS in many areas of the country (9). While CHPS has received massive scientific support right from its inception, there is currently less synthesis of published evidence on the progress of CHPS and how lessons learned can be beneficial to the progress of the programme going forward (9). This study synthesizes scientific evidence on CHPS charting the progress of scientific knowledge in the process.

CHPS is a national strategy to provide healthcare to people in the community and improve primary healthcare in rural areas (10). Since the community’s distance from the closest healthcare facilities limits access to orthodox health care, CHPS was created to help people in remote areas receive basic health services more easily (11). It converts and restructures the primary health care system so that community-based care replaces outreach and facility-based care (12). CHPS also aims to improve efficiency and responsiveness to client needs, as well as to foster effective cross-sector collaboration (13). The CHPS Operational policy by the Ministry of Health stated that CHPS’s overarching strategic goal is to improve the health of Ghanaians by facilitating actions and empowerment at the household and community levels. The CHPS initiative was created to provide a variety of services in health education, health promotion, minor ailment case management, community mobilization for health action, referrals, and home visits (9). In Ghana, there are currently 6,500 CHPS compounds in operation, covering 46% of the country (14).

Akosa (13) in the operational policy also discussed how Ghana Health Service has created an enabling environment for CHPS to operate within the health policies. The CHPS Operational Policy and the National CHPS Policy were used in setting up the CHPS intervention and they serve as guidelines for CHPS operationalization (13). The national health insurance policy framework targets rural communities in Ghana with the intention of achieving universal health coverage (UHC) in line with PHC’s objectives (15, 16). Outpatient care, the majority of inpatient care, including specialty care and most surgeries, maternity care services, including cesarean deliveries, emergency care, and all medications on the NHIA medication list are all covered by the national health insurance policy (17). Additionally, CHPS operates within these broad health policy frameworks; the anti-malarial drug policy, Ghana’s national drug policy, guidelines for strengthening antenatal care policies in Ghana, health care waste management policy and guidelines, traditional medicine policy, and Referral policy and guidelines. A climate of these health policies creates the necessary environment for CHPS to attain its objectives and goals.

CHPS has emerged as the primary strategy for delivering health care in the community, close to the patient. It has proven to be a successful strategy in overcoming barriers to receiving high-quality, inexpensive health care, which has had substantial health consequences. Total expenditure on health as of 2014 was 3.56% Gross Domestic Product (18, 19). According to Yeboah (20), the under-5 child mortality rate decreased from 111 per 1,000 live births in 2003 to 82 per 1,000 live births recorded in 2011. From 1990 to 2010, maternal mortality decreased from 740 per 100,000 live births to 350 per 100,000 live births, and from 2007 to 2017, maternal mortality was recorded at 310 per 100,000 live births. Between 2010 and 2011, the percentage of OPD visits by clients with national health insurance rose from 55.81 to 82.11%. OPD attendance *per capita* rose from 0.98 in 2010 to 1.07 in 2011, with CHPS accounting for about 5% of all OPD visits nationwide (20). In 2016, the average life expectancy for males and females at birth was 62/64 (18, 19). The safe motherhood indicators for the three years under review—92.1% in 2009, 93.3% in 2010, and 94.4% in 2011—show fairly consistent ANC coverage. From 45.6% in 2009, 49.5% in 2010, and 52.2% in 2011, the national rate of skilled delivery has improved steadily (20).

In Ghana, a comprehensive effort to reorganize services led to the establishment of community-based primary healthcare. The Community-based Health Planning and Services (CHPS) initiative aimed to shift primary healthcare services from subdistrict health facilities to more readily accessible community settings (2, 3, 21). The core objectives of the CHPS approach include enhancing equity in basic health service access, fostering collaboration and partnerships across sectors in service delivery, and improving efficiency and responsiveness to clients’ needs.

The CHPS initiative, developed and tested by the Navrongo Health Research Centre, unfolded in successive phases starting in 1994. It was officially launched as a national policy in 1999 and implemented as a nationwide programme in 2000. The initiative underwent a four-phase launch, beginning with a pilot in three villages to formulate effective strategies. Subsequently, a factorial trial demonstrated that community-based care could halve childhood mortality within three years. This was followed by a replication experiment to determine suitable activities for the fourth and final phase—national scale-up (15, 16).

This study sought to explore dominant trends and research themes on Community-based Health Planning and Services. The general objective was to systematically review published scientific evidence on Community-based Health Planning and Services. Specifically, the study seeks to identify, evaluate, and summarize the findings of all relevant individual studies of CHPS and to generate a comprehensive overview of scientific evidence on CHPS.

Four decades after the implementation of Primary health care in Ghana, it was expected that Ghana would have attained UHC by the year 2022 (22). However, there is evidence to show that prevailing circumstances would not make it possible to achieve UHC even by the year 2030 irrespective of the numerous scaling-up interventions and CHPS coverage expansions (23). There are still problems with coverage, affordability, and accessibility of CHPS in rural areas. Also, challenges like inadequate logistics, inadequate skilled personnel, poor documentation, and poor access and delivery services interdict attaining UHC by the year 2030 (24).

Many Ghanaians have received Primary Health Care as a result of CHPS coverage expansions and scaling-up interventions, and it is estimated that Ghana will achieve UHC if current trends continue (22). However, its difficulties may jeopardize this goal (24).

Pervasive challenges of CHPS have been reported nationwide irrespective of all the reported successes. These challenges have been given less attention and as it stands, there has not been any comprehensive understanding of how the reported challenges may systematically affect the implementation of CHPS across the nation (25). This deficit in knowledge may explain the growth atrophy associated with CHPS for some time now. A review of published evidence on CHPS has never been conducted before at the time of this study and as a result, it might be difficult to track the progress of CHPS. This could have consequences such as implicit research bias in the study and difficulty finding CHPS knowledge gaps. It is therefore imperative for a review to be conducted on CHPS. This study seeks to analyze published literature and evidence on Community-based Health Planning Services.

This study is the first of its kind to review and examine reported CHPS literature as at the time it was conducted. The research will provide a systematic analysis of the existing evidence-based CHPS literature. Popular issues, strengths, shortcomings, and deviations will be defined and analyzed, which will help to make informed choices regarding community-based health planning and services.

## Methodology

We carried out a systematic review of Community-Based Health Planning and Services (CHPS) studies conducted in Ghana. In conducting the systematic review, we adhered to the Preferred Reporting Items for Systematic Reviews and Meta-Analyses (PRISMA) guideline to ensure a rigorous and transparent methodology. The study aimed to explore thematic areas within the published literature and assess the progress of CHPS across different regions in Ghana. Comprehensive searches were performed on prominent databases such as Google Scholar, PubMed, and Scopus to identify relevant studies. The Boolean method search query employed was ((Community-Based Health Planning and Services) OR (CHPS) AND (Ghana)). The inclusion and exclusion criteria were pre-determined to narrow down the selection to peer-reviewed papers specifically focusing on CHPS in Ghana, with a publication timeframe between the years 2000 and 2021. Only studies meeting the criteria were considered, ensuring that the chosen articles were directly relevant to the research objectives. The research team (ABA, HB, HA, and AA) convened regular meetings to facilitate thorough discussions and ensure a uniform interpretation and application of the predefined inclusion and exclusion criteria. Subsequently, a diligent examination of eligible studies was undertaken to identify and eliminate any duplicate entries. To mitigate potential selection bias, two investigators (ABA and HB) independently screened the titles and abstracts of the selected articles. Discrepancies that arose during this initial phase were meticulously addressed through collaborative discussions until a consensus was reached. For the evaluation of full-text articles, a similar approach was adopted, with two reviewers (ABA and AA) independently assessing eligibility. Any disagreements between reviewers at this stage were resolved through the involvement of a third reviewer (HA), ensuring a comprehensive and rigorous review process while minimizing the risk of bias. Data extraction and management were conducted using Microsoft Excel, facilitating systematic organization and analysis of the retrieved information. A thematic analysis approach was individually conducted by the team members to synthesize findings. This involved identifying dominant and recurring topics across the literature. The findings were collated, organized, and structured into cohesive themes and interpreted to give the final results.

### Ethical clearance

Ethical approval was sought from the University of Health and Allied Sciences Research Ethics Committee (UHAS-REC), Ho before the start of the study. The protocol identification number of the research is UHAS-REC A.12 [128] 20–21.

### Institutional permission

Institutional permission was sought from the Department of Epidemiology and Biostatistics from the University of Health and Allied Sciences. A clearance letter was received from the UHAS-REC.

### Covid-19 precautionary measures

Secondary data was used in this study therefore no Covid-19 precautionary measures were taken.

## Results

One hundred and thirty-nine (139) publications were selected from Google Scholar, PubMed, and Scopus databases. Twenty-three (23) publications were discovered to be duplicates, and the non-duplicates were one hundred and four (104) publications. Seventy-two (72) publications qualified after the abstract screening. A full-text screening was carried out and fifty-nine (59) publications were selected. The sample size of the study was fifty-nine (59) publications. Supplementary Figure S1 describes the selection process of the studies included in this review. Most of the studies targeted the national level. The sub-district level had the least number of publications targeting it. The papers targeting the community level were also few. Twenty (20) studies targeted the national level, eighteen (18) studies targeted the regional level, sixteen (16) studies targeted the district level, two (2) studies targeted the sub-district level, and three (3) studies targeted the community level. Supplementary Figure S2 describes the various levels the papers targeted. The selected studies were published between the years 2000 to 2021. The year 2017 and 2019 had the highest number of publications on CHPS with seven (7) publications each. The years 2005, 2006, and 2008 had the least publications with only one (1) publication on CHPS. From 2008 to 2012, there was a slopelike increase in the number of publications. The rate of publication was steady from 2012 to 2019, but from 2019 to 2021, there has been a declining decrease in publications. Supplementary Figure S3 describes the year of publication trend.

Many frameworks were identified in the review. Most studies applied the WHO health system framework in their study. The Framework of gender relations was also utilized in some studies. Other frameworks reported included the theory of capital, the Analytical framework, the Consolidated Framework for Implementation Research, and the Reach, Effectiveness, Adoption, Implementation, and Maintenance framework. Some behavioral frameworks used in the studies included Andersen and Newman’s behavioral model of health service utilization, Political Ecology of Health, and Social Psychology of Participation. Most of the studies were qualitative research. The study population comprised community health workers, community health workers, community members, political members, and other CHPS stakeholders. Supplementary Table S1 summarizes the characteristics of the studies.

### Thematic analysis

#### Birth attendants

Birth attendant services of CHPS encompass a range of providers, including traditional birth attendants and midwives. Various factors influence skilled birth, such as religious affiliation, care visits, education, parity, partner’s education level, region of residence, and maternal age. Additionally, other important factors which influence birth attendant services are birthplace of clients, wealth level, and the skill of the medical staff. The proficiency of birth attendants can attract pregnant women to seek care at their chosen delivery location. The community places trust in the midwives at CHPS compounds, where they fulfill roles like skilled delivery, health education, counseling, and antenatal care services. Despite positive outcomes of birth services, there is a belief that enhancing the effectiveness of antenatal care attendance requires community engagement, volunteerism, and collaboration with local authorities and community members.

#### Care continuity, clients satisfaction and sustainability of CHPS

Ensuring continuity of care is vital within the CHPS framework. Recognizing the importance of community involvement in selecting health volunteers has been acknowledged as a factor promoting this continuity. Enhancements in both ANC (antenatal care) and PNC (postnatal care) services, coupled with extensive outreach coverage, also contribute to improving care continuity. Patient satisfaction plays a significant role in maintaining continuity of care, and a scale measuring this satisfaction includes categories such as happy, good, satisfied, unsatisfied, and cordial. Encouragingly, a substantial number of customers have expressed their contentment with CHPS services, indicating a positive trend in maintaining continuity of care with the majority rating their satisfaction as happy, good, and satisfied. The sustainability of the CHPS programme will necessitate a high capacity for the programme to maintain its operations, effectiveness, and community support. The programme’s ability to adapt to changing health priorities and integrate into existing healthcare infrastructure contributes to its long-term viability. The CHPS programme’s sustainability is also dependent on its alignment with broader health policies at the national and global levels.

#### Resources and support

Essential elements needed for CHPS advancement encompass human resources, donor assistance, financial backing, equipment, technology, and logistical support. Regrettably, most CHPS facilities currently face shortages in these critical resources. Challenges include insufficient infrastructure, inflexible resource availability, inadequate financial planning and budgeting, and logistical obstacles for Community Health Volunteers (CHV) in terms of necessary tools like torches, raincoats, bicycles, and boots. To achieve effectiveness, CHPS requires comprehensive support, particularly from the State and the local community.

#### Family planning and low involvement of men in health

The family planning service integrated into CHPS aims to regulate childbirth and prevent pregnancies. Despite this, some women face resistance from their partners, particularly men who are opposed to their wives adopting any family planning methods. This opposition is predominantly linked to cultural norms, negative attitudes toward family planning techniques, gender roles, and superstitious beliefs. Nevertheless, health interventions addressing these factors have led to significant advancements in men’s engagement with healthcare. Furthermore, it is observed that fewer men are formally recognized in roles such as nurses or community workers, which are predominantly occupied by women.

#### Community engagement and participation

The community plays a substantial role in shaping the design and needs analysis of CHPS. The programme gained acceptance primarily due to its healthcare benefits. The established CHMT represented the interests of the entire community, inclusive of all its groups, and managed CHPS without external interference. Contributions, both monetary and in-kind, were accepted, with local residents providing labor and supplies. Successful collaboration with other local organizations was facilitated by the CHMT. The involvement of traditional leaders significantly influenced participation, and a community’s active engagement in CHPS was more likely when there was a sense of social capital and communal value. Community participation was shaped by the beliefs and perceptions of community members, leaders, stakeholders, and experts. The commitment to volunteering and active involvement was solidified through community engagement, fostering a sense of community pride, exceptional services, contentment, appreciation, satisfaction, hard work, and altruism. CHPS has garnered high community acceptance, with reports indicating appreciation for health services provided during home visits and improved access to medical facilities.

#### Referral system

Several factors adversely affect the functionality of the CHPS referral system, with one major impediment being endemic poverty, especially in rural areas. The referral process is hindered by challenges such as poor road conditions, exorbitant transportation costs, and impassable roads. In certain rural areas, vehicles operate only on market days, which occur once or twice a week. Consequently, transportation difficulties often lead to non-compliance with referrals. Individuals encountering referral challenges may often resort to traditional remedies instead of seeking care at medical facilities.

#### Challenges of CHPS

##### Health economic

The high expense of medical care and the deficiency of transportation and infrastructure present the CHPS programme with serious financial challenges. Staffing, infrastructure, and the availability of necessary medical supplies are all impacted by the lack of funding that makes it difficult to establish and maintain the CHPS programme. Without strong government backing, CHPS faces more financial hardships, which make them heavily dependent on outside funding and make their integration into the larger national health system more difficult. Insured clients of Ghana’s NHIS who seek health care in CHPS facilities make out-of-pocket payments for consultations and drugs covered by the scheme. The out-of-pocket payments are largely attributed to the lack of drugs at the CHPS facilities. Clients are also charged consultation fees to cover administrative costs. These economic inefficiencies affect CHPS’s overall functionality and service delivery. These inefficiencies include problems with transportation, supply chain management, and inadequate funding. To make matters worse, these challenges affect the ability of the CHPS programme to remain economically viable and affordable.

##### Social

The lack of public knowledge about CHPS’s interventions and activities is a major social challenge. The community’s involvement and participation in healthcare programmes are also impacted by ignorance. When there is ineffective community collaboration among the people, it leads to social barriers that can impede resource utilization and reduce the overall efficacy of the CHPS initiative. Furthermore, residents who choose not to use CHPS services in favour of alternative therapies impact the programme’s efficacy and reach, gradually limiting the recognition of CHPS within communities, which further reduces its utilization. Communication barriers, such as language barriers and misunderstandings also influence effective healthcare delivery and community engagement. When the role of CHVs are undermined by the lack of appreciation they receive, it affects their motivation and efficacy in community health initiatives.

##### Anthropology

From an anthropological perspective, CHPS’s use of inadequate personnel and logistics could undermine community confidence in the healthcare system. This is further increased by difficulties with information storage, use, education, and intervention. These issues affect how health-related knowledge is shared within communities. Anthropologists have also observed that the physical distance between CHPS compounds—particularly in rural areas—influences community members’ inclination to seek medical attention. Inconsistent training and low motivation among CHOs and CHVs is another challenge that influences how communities view healthcare providers. These challenges are further compounded by the fact that the lack of skill development programmes hinders the professional development and efficacy of healthcare providers operating within the CHPS framework.

#### Overcoming CHPS challenges

To address its challenges, CHPS requires sustainable, cost-effective, and scalable mechanisms. The range of CHPS services should be broadened, with a focus on providing essential emergency services. The successful implementation of healthcare in every community necessitates the active involvement of traditional leaders and grassroots activists. Essential components for CHPS include resource availability, intensified outreach efforts, the provision of cost-free maternal health services, and improved referral services. Achieving expanded coverage and mobilizing resources across multiple sectors are imperative. CHPS personnel need training in logistics management, data management, conflict resolution, managerial skills, and leadership. Essential prerequisites include field training in areas such as integrated management of neonatal and pediatric illnesses, neglected tropical disease management, and Integrated Disease Surveillance and Response (IDSR). Supervision should be in place to provide technical assistance and training services, supporting personnel in their roles.

#### Policy implication

The identified challenges surrounding the Community-Based Health Planning and Services (CHPS) programme underscore the imperative need for targeted policy interventions at both national and global levels. To effectively scale up the CHPS programme, policymakers must prioritize addressing the root causes of challenges such as inadequate transportation, limited public awareness, and high healthcare costs. Allocating resources for comprehensive training programmes that encompass logistics management, conflict resolution, and leadership skills is crucial for building a resilient workforce of Community Health Officers (CHOs) and Volunteers (CHVs). Moreover, aligning CHPS with national and global health policies requires strategic collaboration with traditional leaders, grassroots activists, and multi-sectoral stakeholders to ensure community engagement and resource mobilization. Policy interventions should focus on expanding the scope of CHPS services, with an emphasis on essential emergency and maternal health services. Sustainable funding mechanisms and strengthened cold chain systems are essential for overcoming logistical challenges and ensuring the availability of quality healthcare. Ultimately, a well-supported and effectively implemented CHPS programme aligns with broader health policy goals, contributing to the achievement of universal health coverage and improved health outcomes for both rural and urban populations.

## Discussion

Community health workers have been identified as critical players in achieving universal health coverage and supplementing efforts to improve primary healthcare delivery in low-and middle-income countries (26). In a study conducted by Kweku et al., health workers shared their perspectives on the barriers to CHPS implementation in Ghana and how these barriers can be overcome. They believed that some health-system challenges include a lack of referrals, a lack of proper community entry and engagement, a lack of essential logistics, a long distance between CHPS compounds and communities, and insufficient funding.

It is commonly acknowledged that healthcare accessibility plays a crucial role in the overall health system (27). Nonetheless, it is impacted by variables like racial or ethnic background, language, disability, mobility, distance to medical care, and the quantity of medical professionals in a given area (27). According to Agbenyo et al. (28), additional factors could be the burden of care at home, customs that prohibit women from scheduling early ANC visits, the expense of evaluating medical care, and public mistrust of healthcare institutions. The two main obstacles to using and accessing healthcare facilities are thought to be transportation and distance. A shorter distance to medical facilities might encourage walking (27). The issue of transportation arises from the inadequate road infrastructure, compounded by erosion and the muddy conditions of roads, particularly exacerbating mobility challenges during the rainy season (27). Reports highlight significant health concerns among rural residents, with a particular emphasis on the potential survival challenges faced by patients in peri-urban areas during health emergencies due to the lack of accessibility to healthcare facilities (29).

Several researchers have conducted assessments to gauge the impact of the Community-based Health Planning and Services (CHPS) initiative. Johnson et al. (30) found that establishing CHPS compounds near health facilities enhances healthcare accessibility. Concerning skilled birth care, areas with health facilities and CHPS facilities within 8 km demonstrate a 16% higher utilization of skilled birth care compared to areas where only a health facility is present within the same distance (30).

In a study by Kolbila (31), various benefits of CHPS implementation were underscored. Improved physical access to healthcare was identified as a prominent advantage, leading to enhanced access to antenatal care (ANC) services, resulting in increased attendance by women at CHPS compounds. Furthermore, the strategic placement of CHPS compounds at the community level was highlighted for its positive impact on accessing and utilizing health services, both at clinics and hospitals. Beyond healthcare, CHPS compounds also serve social purposes by acting as community centers and public spaces, fostering social interaction and providing a venue for various community activities (31). Additionally, CHPS contributes to community development by imparting skills and knowledge to residents and serving as an institution for educating individuals about essential healthcare practices. Woods (32) supported this perspective, affirming that the presence of health services in rural areas has contributed to increased community sustainability and development.

According to Nwameme et al. (33), CHOs consider the supervisory activities carried out by CHPS coordinators at the district and regional levels to be insufficient. In their attempt to accompany the CHOs on regular home visits to supervise their activities in the community and offer help where needed, the supervisors face a variety of challenges, including transportation issues, harsh terrain, and a lack of staff strength (33). Health workers believe that professional capacity development and ongoing community engagement are avenues for improving CHPS programme implementation. Furthermore, CHPS communities located near healthcare facilities improve access to care (30).

Community-based health volunteerism plays a vital role in numerous health systems and initiatives, contributing to the promotion and delivery of diverse health interventions and disease surveillance. The shortage of formal healthcare workers has necessitated the engagement of Community-Based Health Volunteers (CBHV) to provide essential healthcare services, particularly in rural and overlooked communities. Over time, a persistent challenge has been retaining these volunteers and sustaining their activities (34).

These volunteers encounter various obstacles, including a lack of respect and support from community members, as well as a dearth of incentives and essential supplies such as raincoats, torchlights, Wellington boots, and bicycles for transportation, which are crucial for facilitating their movement. These challenges have proven discouraging for volunteers (34). Additionally, financial, logistical, and telecommunication challenges, coupled with a lack of recognition and cooperation from community members, insufficient motivation, and an absence of regular skill development training programmes for Community Health Management Committee (CHMC) members serving as traditional birth attendants (TBAs), have been identified as some significant hurdles in CHMC volunteerism. Nonetheless, the primary drivers of their work as health volunteers are their desire to assist the community and their status and recognition as medical professionals (34). Verbal praise, direction, and social gatherings boost volunteers’ spirits and motivate them to work harder, according to a Dil et al. study. Value, comprehension, and protective roles were asserted by Kweku et al. as the primary drivers of CHMC service.

Also because nurses are primarily female and nursing faculties are frequently predominately composed of women, gender bias and role stereotyping occur in nursing education programmes (35). Men are not allowed to hold certain positions or participate in certain types of training. For instance, midwifery credentials may be required to apply for some senior nursing positions (such as director of nursing), but men are prohibited from pursuing midwifery training in a number of nations, making them ineligible for these roles (36). Even though they believe they are given more responsibilities and are occasionally passed over for promotions, the majority of men in the field do not wish to quit (37). According to a 2009 study by Malcher (38), men’s involvement in healthcare is crucial for social justice, as well as for enhancing present health outcomes and expanding potential health advantages.

## Conclusion

The principal approach to delivering healthcare close to the patient in the community is now Community-based Health Planning and Services (CHPS). In order to address obstacles to receiving high-quality, reasonably priced healthcare that have a substantial impact on health, it has proven to be a useful tool. Since its introduction, many good health outcomes have been documented. It acts as the first point of call for primary healthcare and has become the primary method of delivering close-to-client healthcare in the community.

## Limitation

The study has several limitations that should be considered. First, the study relied solely on peer-reviewed published literature, ignoring grey literature and unpublished studies. Also, the study’s timeframe (2000–2021) may make it less reflective of recent developments in the rapidly changing healthcare landscape. The heterogeneity of challenges across regions or communities was not thoroughly investigated, and a comparative analysis could lead to a better understanding of regional variations in CHPS implementation. Addressing these limitations in future research efforts would help to provide a more comprehensive and nuanced understanding of CHPS in Ghana.

## Future research direction

Prospective research avenues for addressing the effects and difficulties of the CHPS programme ought to promote inclusivity through the integration of varied viewpoints from community members, healthcare providers, and policymakers. Extending the temporal range of our examination to include past research and historical information will yield a thorough comprehension of the programme’s development and enduring effects. Furthermore, incorporating reports from non-governmental organizations and government agencies into the grey literature is essential to obtaining a useful understanding of the CHPS programme. Comparing this model with similar community-based healthcare models from other nations will provide useful baselines and point out possible areas for development. Also, understanding the varying effects on men and women requires a gender analysis, which clarifies gender dynamics in healthcare delivery and access. To make sure the programme is financially sustainable, it is essential to investigate health financing options, including health insurance’s role. To determine whether the programme can be sustained, a careful examination of human resource management is necessary, with a focus on the hiring, development, and retention of community health workers. A discussion on the role of innovation and technology, such as telemedicine and mobile health, will highlight opportunities for improving healthcare delivery within the CHPS framework. Quality assurance mechanisms, which include monitoring, evaluation, and feedback, should also be investigated.

## Data availability statement

The original contributions presented in the study are included in the article/Supplementary material, further inquiries can be directed to the corresponding author.

## Author contributions

ABA: Writing – original draft, Conceptualization, Data curation, Formal analysis, Methodology. HB: Writing – review & editing, Data curation, Methodology. HA: Writing – review & editing, Supervision. AA: Supervision, Writing – review & editing.
