# Supplementary material for: Community-based Health Planning and Services programme in Ghana: a systematic review
Source: Front Public Health. 2024 Mar 5;12:1337803. doi: 10.3389/fpubh.2024.1337803 (PMC10948426; doi:10.3389/fpubh.2024.1337803)
Supplement: Supplementary file 1 [file Data_Sheet_1.docx]

**Supplementary Material**

1. **Supplementary Figure 1:** Prisma flow diagram. Presentation of the procedure of literature search and selection with the number of articles at each stage.


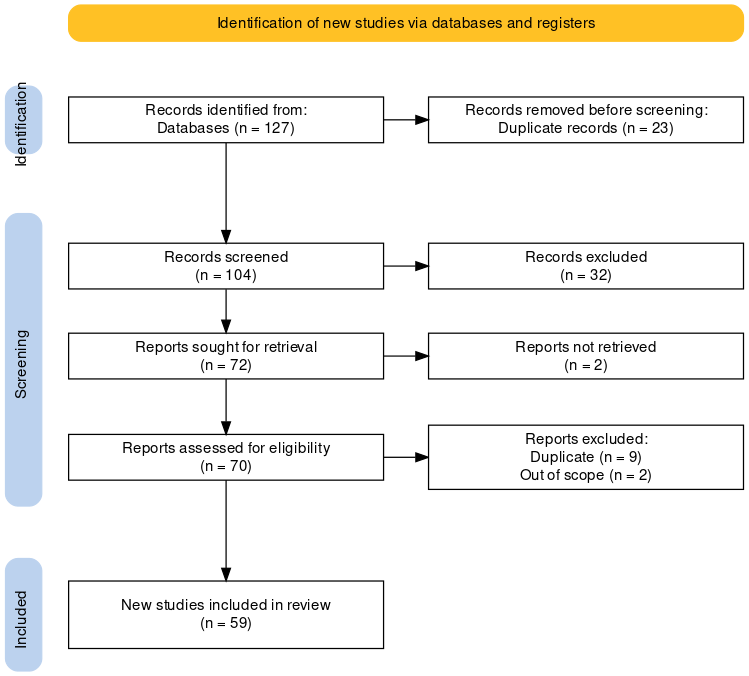


1. **Supplementary Figure 2:** Publications targeting the various health levels.
2. **Supplementary Figure** 3**:** Analysis of Year of Publication
3. **Supplementary Table 1:** Study Characteristics

|  | Title | Authors | Journal | Year | Study design | Population | Sample Size | Region | Main subject |
| --- | --- | --- | --- | --- | --- | --- | --- | --- | --- |
| 1 | The Ghana essential health interventions program: A Plausibility Trial of the impact of Health Systems Strengthening on maternal & child survival | John Koku Awoonor-Williams, Ayaga A Bawah, Frank K Nyonator, Rofina Asuru, Abraham Oduro, Anthony Ofosu, James F Phillips | BMC Health Services Research | 2013 | A plausibility trial of the impact of health systems strengthening on maternal & child survival | Community volunteers, community health nurses, grassroots political stakeholders | 6,300 | Kassena Nankana District of the Upper East Region (UER), Ghana's poorest region | The main subject of the paper is the Ghana Essential Health Intervention Project (GEHIP), which is a plausibility trial of strategies for strengthening the Community-based Health Planning and Services (CHPS) model, especially in the areas of maternal and newborn health, and generating the political will to scale up the program with strategies that are faithful to the original design |
| 2 | A qualitative analysis of the effect of a community-based primary health care programme on reproductive preferences and contraceptive use among the Kassena-Nankana of northern Ghana | Maxwell Ayindenaba Dalaba, Allison E Stone, Abigail R Krumholz, Abraham R Oduro, James F Phillips, Philip B Adongo | BMC Health Services Research | 2016 | Qualitative study | The population of the Kassena Nankana East and West Districts of Ghana's Upper East Region | 152000 | Kassena Nankana East and West Districts of Ghana's Upper East Region | The main subject of the paper is the effect of a community-based primary health care programme on reproductive preferences and contraceptive use among the Kassena Nankana of northern Ghana |
| 3 | Challenges to achieving universal health coverage through community-based health planning and services delivery approach: a qualitative study in Ghana | Abraham Assan, Amirhossein Takian, Moses Aikins, Ali Akbarisari | BMJ | 2019 | Qualitative Study | Policy makers, managers of CHPS compound and health centres, politicians, academics, health professionals, technocrats, and community health management committee members. | 67 | Ghana | The main subject of the study was to investigate ways to provide sustainable community health financing and resource mobilisation in Ghana |
| 4 | Can she make it? Transportation barriers to accessing maternal and child health care services in rural Ghana | Nasung Kilian, Atuoye, Jenna Dixon, Andrea Rishworth, Sylvester Zackaria Galaa, Sheila A Boamah, Isaac Luginaah | BMC Health Services Research | 2015 | Qualitative Study | Males and females aged between 18 and 70 | 85 | Upper West Region of Ghana | The main subject of the paper is the challenges of transportation in accessing maternal health care services in rural Ghana |
| 5 | Catalyzing the scale-up of community-based primary healthcare in a rural impoverished region of northern Ghana | John Koku Awoonor-Williams, James F Phillips, Ayaga A Bawah, J K Awoonor-Williams | International Journal of Health Planning and Management | 2016 | Controlled plausibility trial | - | - | Ghana | The main subject of the paper is challenges and deficiencies in community based primary healthcare in a rural impoverished region of northern Ghana |
| 6 | Community utilisation and satisfaction with the community-based health planning and services initiative in Ghana: a comparative study in two system learning districts of the CHPS+ project | Margaret Kweku, Hubert Amu, Martin Adjuik, Yayra Fortress, Aku, Emmanuel Manu, Elvis Enowbeyang Tarkang, Joyce Komesuor, Geoffrey Adebayor Asalu, Norbert Ndaah Amuna, Laud Ampomah Boateng, Justine Sefakor Alornyo, Roland Glover, Ayaga A Bawah, Timothy Letsa, John Koku, James F Phillips, John Owusu Gyapong | BMC Health Services Research | 2020 | A community based descriptive study | Adults | 1008 | Central Tongu and Nkwanta South | The main subject of the paper is community utilisation and satisfaction with the community based health planning and services initiative in Ghana |
| 7 | Chronic non-communicable diseases and the challenge of universal health coverage: insights from community-based cardiovascular disease research in urban poor communities in Accra, Ghana | Ama De-Graft Aikins, Mawuli Kushitor, Kwadwo Koram, Stella Gyamfi, Gbenga Ogedegbe | BMC Public Health | 2014 | Community based cardiovascular disease research | Urban poor communities in Accra, Ghana | - | Urban poor communities in Accra, Ghana | The main subject of the paper is the impact of cardiovascular diseases on primary healthcare services in urban poor communities in Accra, Ghana, and the challenge of achieving universal health coverage in the context of the primary healthcare system in low and middle-income countries. The paper discusses the implications of this burden on health systems and communities and identifies challenges related to financing healthcare, strengthening the health workforce, and addressing issues of equity and rights |
| 8 | Community-Based Health Planning and Services Plus programme in Ghana: A qualitative study with stakeholders in two Systems Learning Districts on improving the implementation of primary health care | Margaret Kweku, Hubert Amu, Adam Awolu, Martin Adjuik, Martin Amogre Ayanore, Emmanuel Manu, Elvis Enowbeyang Tarkang, Joyce Komesuor, Geoffrey Adebayo Asalu, Yayra Fortress, Aku, Nuworza Kugbey, Fidelis Anumu, Laud Ampomah Boateng, Justine Sefakor Alornyo, Roland Glover, Timothy Letsa, Ayaga A Bawah, Nicholas S Kanlisi, John Koku Awoonor-Williams, James F Phillips, John Owusu Gyapong | PLoS ONE | 2020 | Qualitative study | Adults | 60 | Volta Region of Ghana | The main subject of the study was to explore the challenges, capacity development priorities, and stakeholder perspectives on improving the Community Based Health Planning and Services (CHPS) concept in Ghana and identifying challenges in the implementation of primary health care in Ghana |
| 9 | Accelerating reproductive and child health programme impact with community-based services: the Navrongo experiment in Ghana | James F Phillips, Ayaga A Bawah, Fred N Binka, | Bulletin of the World Health Organization | 2006 | Plausibility design | - | - | Kassenan Nankana District | The main subject of the paper is the impact of deploying nurses and volunteers in villages to expand health services and accelerate reproductive and child health program impact |
| 10 | Repositioning community-based family planning in Ghana: A case study of Community-based Health Planning and Services (CHPS) | Stephen Ntsua, Frank K Nyonator, Placide Tapsoba, Gloria Quansah, Gloria Quansah Asare, Noah Abisola, F Esantsi | Social and behavioral science reseaarch | 2012 | A case study, qualitative focus group discussion | DCE, district directors, chps coordinators | 28 | Komenda, Biabiani | The main subject of the excerpt is Training and Supervision in the context of repositioning community based family planning in Ghana |
| 11 | Evaluating the Impact of the Community-Based Health Planning and Services Initiative on Uptake of Skilled Birth Care in Ghana | Amoako Fiifi, Johnson, Faustina Frempong-Ainguah, Zoe Matthews, Andrew J P Harfoot, Philomena Nyarko, Angela Baschieri, Peter W Gething, Jane Falkingham, Peter M Atkinson | PLoS ONE | 2015 | Survey Data from the 2003 and 2008 Demographic and Health Survey | Women and men aged 15-49 years and 15-59 years in rural communities in Ghana | 4,349 births | Ghana | The main subject of the paper is the Community-Based Health Planning and Services Initiative (CHPS) and its impact on the uptake of skilled birth care in Ghana |
| 12 | Guiding the Ghana Community-based health planning and services approach to scaling up with qualitative systems appraisal. | F Nyonator, Tanya C Jones, Robert A Miller, James F Phillips, John Koku | Sage | 2003 | Commentary | - | - | Ghana | The main subject of the article is the Community based Health Planning and Services (CHPS) Initiative and the Qualitative Systems Appraisal (QSA) of factors explaining why CHPS is implemented in some districts, but stalled in others |
| 13 | Quality of Antenatal Care Services in the Birim North District of Ghana: Contribution of the Community-Based Health Planning and Services Program | Sharon Naariyong, Krishna C Poudel, Mosuir Rahman, Junko Yasuoka, Keiko Otsuka, Masamine Jimba, Á K C Poudel, Á M Rahman, Á J Yasuoka, Á K Otsuka, Á M Jimba | Maternal and Child Health Journal | 2012 | Survey | Mothers aged 15-49 years who had at least 1 child within 18 months of age, resided in the district for at least 2 years before the data collection period | 600 | Birim North District of Ghana | The main subject of the paper is to assess the quality of care in Birim North District looking at the CHPS programme. |
| 14 | The Ghana Community-based Health Planning and Services Initiative: Fostering Evidence-based Organizational Change and Development in a Resource-constrained Setting | Frank K Nyonator, James F Phillips, Tanya C Jones, Robert A Miller | Population Council | 2003 | Review | - | - | Ghana | The main subject of the paper is the Ghana Community based Health Planning and Services Initiative and the usage to foster change and development. |
| 15 | Managing for Quality in Health Care: Quality Improvement Issues in Ghana's Community-based Health Planning and Services Concept | Reuben K Esena | Journal of Scientific and innovation research | 2013 | Review | - | - | Ghana | The paper explored quality improvement issues in CHPS and how to manage them in healthcare. |
| 16 | Noso-politics' and Japanese Development Assistance : The Scaling-up of Community-based Health Planning and Services (CHPS) in Ghana | Kweku Ampiah | Kansai national institute of informatics | - | Review | - | - | Upper West region | The paper explores Japan’s contribution to the provision of primary healthcare in Ghana through the Community-based Health Planning and Service （CHPS） strategy |
| 17 | Challenges to the utilization of Community-based Health Planning and Services: the views of stakeholders in Yendi Municipality, Ghana | Bougangue Bassoumah, Andani Mohammed Adam, Martin Nyaaba Adokiya | BMC Health Research | 2021 | Qualitative study | The population of the Yendi Municipality | 34 | Yendi Municipality | The main subject of the paper is the challenges faced in the utilization of Community-based Health Planning and Services (CHPS) and the National Health Insurance Scheme (NHIS) in Yendi Municipality, Ghana |
| 18 | Factors facilitating and constraining the scaling up of an evidence-based strategy of community-based primary care: Management perspectives from northern Ghana | Abigail R Krumholz, Allison E Stone, Maxwell A Dalaba, James F Phillips, Philip B Adongo | Global Public Health | 2015 | Qualitative Study | Adults | 12 | Northern Ghana | The main subject of the paper is community-based primary care strategy perspective by stakeholders |
| 19 | Assessing participation in a community-based health planning and services programme in Ghana | Leonard Baatiema, Morten Skovdal, Susan Rifkin, Catherine Campbell | BMC Health Services Research | 2013 | Qualitative Study | The total population of Wa Municipal is 116,460 | 19 | Wa Municipal of the Upper West Region of Ghana, West Africa | The main subject of the interviews and focus group discussions was community participation in a health planning and services programme in Nachanta, Ghana |
| 20 | Integrated community case management and community-based health planning and services: a cross sectional study onthe effectiveness of the national implementation for the treatment of malaria, diarrhoea and pneumonia. | Blanca Escribano Ferrer, Jayne Webster, Jane Bruce, Solomon A Narh-Bana, Clement T Narh, Naa-Korkor Allotey, Roland Glover, Constance Bart-Plange, Isabella Sagoe-Moses, Keziah Malm, Margaret Gyapong | BMC | 2016 | Household Survey | The study population were carers of children under 5 years of age, who had fever, cough and or diarrhoea in the last 2 weeks prior to the interview | 1356 | Ghana | The main subject of the paper is the effectiveness of the national implementation of integrated community case management and community based health planning and services for the treatment of malaria, diarrhoea, and pneumonia in Ghana |
| 21 | Motivations and Challenges of Community-Based Surveillance Volunteers in the Northern Region of Ghana | Yasemin Dil, Daniel Strachan, Sandy Cairncross, • Andrew, Seidu Korkor, Zelee Hill, Á D Strachan, Á Z Hill, A S Korkor | Journal of community health | 2012 | Qualitative Study | 28 CBSVs, 12 zonal coordinators, nine Ghana Health Service (GHS) sub-district level staff, ten GHS district level staff and two GHS regional level staff in the administrative capital | 52 | Northern Region of Ghana | The main subject of the paper is the use of Community Health Workers (CHWs) and volunteers in low-income countries and some challenges and motivations required for their work. |
| 22 | The association between health insurance status and utilization of health services in rural Northern Ghana: evidence from the introduction of the National Health Insurance Scheme | Philip Ayizem Dalinjong, Paul Welaga, James Akazili, Anthony Kwarteng, Martin Bangha, Abraham Oduro, Osman Sankoh, Jane Goudge | Journal of Health, Population and Nutrition | 2017 | Cross sectional household survey | People of Kassena Nankana districts (East and West) | 55,992 | Kassena Nankana districts (East and West) of the Upper East region of Ghana | The main subject of the paper is the National Health Insurance Scheme (NHIS) in Ghana and its association with healthcare utilization. |
| 23 | Contribution of Community Based Health Planning Service in Maternal Health Service Delivery in the Tamale Metropolitan Area, Ghana | Mavis M Begohn, Dina Adei, Isabella S Mireku |  | 2016 | Cross sectional | Female population for each of the selected zones from 2010 to 2014. | 395 | Tamale Metropolitan Area in the northern region of Ghana | The main subject of the paper is the contribution of Community Based Health Planning Service in maternal health service delivery in the Tamale Metropolitan Area, Ghana |
| 24 | Cost of implementing a community-based primary health care strengthening program: The case of the Ghana Essential Health Interventions Program in northern Ghana | Edmund Wedam Kanmiki, James Akazili, Ayaga A Bawah, James F Phillips, John Koku, Patrick O Asuming, Abraham R Oduro, Moses Aikins | PLoS ONE | 2019 | Plausibility trial | - | - | Northern Ghana | The main subject of the paper was a health system strengthening and research program called the Ghana Essential Health Interventions Program (GEHIP) |
| 25 | The child survival impact of the Ghana Essential Health Interventions Program: A health systems strengthening initiative in a rural region of northern Ghana | Ayaga A Bawah, John Koku, Patrick O Asuming, Christopher B Boyer, Sebastian F Achana, James Akazili, James F Phillips | PloS ONE | 2019 | Plausibility design | Population of Upper East region | 8,917 | Ghana's Upper East Region (UER) of northern Ghana | The paper explored the health system strengthening initiative in rural region of northern Ghana |
| 26 | Factors influencing performance of community-based health volunteers' activities in the Kassena-Nankana Districts of Northern Ghana | Samuel Chatio, Paul Welaga, Philip Teg-Nefaah Tabong, Patricia Akweongo | PLoS ONE | 2019 | Cross-sectional design | Volunteers of the Kassena Nankana East District and Kassena Nankana West District of Northern Ghana | 200 | Kassena Nankana East District and Kassena Nankana West District of Northern Ghana | The main subject of the paper is factors affecting the performance of community based health volunteers in the Kassena Nankana Districts of Northern Ghana |
| 27 | Can community health officer-midwives effectively integrate skilled birth attendance in the community-based health planning and services program in rural Ghana? | Evelyn Sakeah, Lois Mccloskey, Judith Bernstein, Kojo Yeboah-Antwi, Samuel Mills | BMC | 2014 | Qualitative Study (IDI) | Health professionals and community stakeholders. | 41 | Kassena Nankana East, Kassena Nankana West, and Bongo Districts of the Upper East Region (UER) of Ghana | The paper assesses the feasibility of and extent to which the skilled delivery program has been implemented as an integrated component of the existing CHPS and documents the benefits and challenges of the integrated program. |
| 28 | Cost-effectiveness analysis of the national implementation of integrated community case management and community-based health planning and services in Ghana for the treatment of malaria, diarrhoea and pneumonia | Blanca Escribano Ferrer, Kristian Schultz Hansen, Margaret Gyapong, Jane Bruce, Solomon A Narh Bana, Clement T Narh, Naa-Korkor Allotey, Roland Glover, Naa-Charity Azantilow, Constance Bart-Plange, Isabella Sagoe-Moses, Jayne Webster | BMC | 2017 | Cost effectiveness analysis | Carers of children under-5 years who had fever, diarrhoea and/or cough in the last 2 weeks prior to the interview | 1296 | Ghana, specifically the Volta Region and Northern region | The main subject of the paper is a cost effectiveness analysis of integrated community case management (iCCM) versus community based health planning and services (CHPS) for the treatment of malaria, diarrhoea, and pneumonia in Ghana |
| 29 | Community-based health insurance and access to maternal health services: Evidence from three West African countries | Kimberly V Smith, Sara Sulzbach, Marty Makinen, Lynne Miller Franco, Sara Bennett, Xinghu Liu, Clara Burgert | Social Science & Medicine (1967) | 2008 | Comparison group design (Household survey) | Users of community-based health insurance | - | Ghana, Mali and Senegal | The main subject of the paper is community based health insurance (CBHI) and its potential to reduce out of pocket expenditures for health services in developing countries, particularly in rural and informal sector households |
| 30 | The influence of the Community-based Health Planning and Services (CHPS) program on community health sustainability in the Upper West Region of Ghana | Hannah Woods, \| Umar Haruna, Irenius Konkor, Isaac Luginaah, Umar Haruna | Wiley Library | 2018 | Qualitative study | Community members and health officials | 33 | Upper West Region (UWR) of Ghana | The main subject of the paper is the influence of social and human capital on community health sustainability in the context of the Community based Health Planning and Services (CHPS) program in the Upper West Region of Ghana |
| 31 | Is there any role for community involvement in the community-based health planning and services skilled delivery program in rural Ghana? | Evelyn Sakeah, Lois Mccloskey, Judith Bernstein, Kojo Yeboah-Antwi, Samuel Mills | BMC Health Services Research | 2014 | Intrinsic case study design with qualitative methodology | Health professionals and community stakeholders. | 29 | Kassena Nankana East (KNE), Kassena Nankana West (KNW), and Bongo districts of UER of Ghana | The main subject of the paper is examining the role of community involvement in the community based health planning and services skilled delivery program in rural Ghana |
| 32 | Factors influencing the uptake of family planning services in the Talensi District, Ghana | Paschal Apanga, Matthew Ayamba Adam | The Pan African Medical Journal | 2015 | Descriptive cross-sectional correlational design | Women aged 15-49 years in the Talensi District, Ghana | 280 | Talensi District, Ghana | The paper aimed to investigate the factors that influence the decision of women in fertility age to go for family planning services. |
| 33 | Using the community-based health planning and services program to promote skilled delivery in rural Ghana: socio-demographic factors that influence women utilization of skilled attendants at birth in Northern Ghana | Evelyn Sakeah, Henry V Doctor, Lois McCloskey, Judith Bernstein | BMC Public Health | 2014 | Cross-sectional household Survey | Women | 369 | Kassena-Nankana East(KNE), Kassena-Nankana West (KNW), and Bongo Districts | The main subject of the paper was using CHPS to promote Skilled birth attendants |
| 34 | The role of community-based health services in influencing postnatal care visits in the Builsa and the West Mamprusi districts in rural Ghana | Evelyn Sakeah, Raymond Aborigo, James Kotuah Sakeah, Maxwell Dalaba | BMC Pregnancy and Childbirth | 2018 | Cross-sectional study | Women | 1623 | Builsa and the West Mamprusi Districts of the Upper East and Northern Regions of Ghana | The main subject of the paper was to explore how CHPS influences Postnatal care visit |
| 35 | Scaling down to scale-up: a strategy for accelerating community-based health service coverage in Ghana | John Koku Awoonor Williams, James F. Phillips, Ayaga A. Bawah | Journal of Global Health Science | 2019 | District trial | Community Health Nurses, Community volunteers, DHMT | 32 DHMT | Different sub districts and districts in Ghana | The main subject of the paper was to explore Scale-up to scale-down for accelerating CHPS. |
| 36 | Suicide in Ghana: How Could the Community-Based Health Planning and Service (CHPS) Effectively Contribute to Its Prevention? | Abraham Assan, Moses Aikins, Amirhossein Takian | Iran J Public Health | 2018 | Review | - | 9 articles | Ghana | The paper explored suicide in Ghana and how CHPS can aid in prevention. |
| 37 | Sustaining health intervention programmes in ghana: the role of the ‘community’ in the community-based health planning and services. | Aaron Kwasi Nartey, Esmeranda Manful | Research Gate | 2018 | Mixed method: A total of five Focus Group Discussions and In-depth interviews with eight stakeholders were conducted | Respondents, Stakeholders | 384 | Barekese sub-district of Ashanti Region | The paper explored the role of community in promoting CHPS through sustainable health interventions. |
| 38 | The Architecture of a Software System for Supporting Community-based Primary Health Care with Mobile Technology: The Mobile Technology for Community Health (MoTeCH) Initiative in Ghana | Bruce MacLeod, James Phillips, Allison E. Stone, Aliya Walji, John Koku Awoonor-Williams | Online Journal of Public Health Informatics | 2012 | Commentary | Clientele, Frontline workers, Community health volunteers, | - | Ghana | The main subject of the paper was to explore software systems for promoting CHPS |
| 39 | The Contribution of Community-based Health Planning and Services (CHPS) to Community Sustainability and Health in the Upper West Region of Ghana | Hannah M. Woods | Western Graduate and Postdoctoral Studies | 2016 | Case study | Community health nurses, community health volunteers, community health officers | 44 | Dabo, Varimpere ,Talawona Nadowli, Gbanko, Naro, Kpazie in Upper West region | The main subject of the paper was to explore contribution of CHPS in community sustainability |
| 40 | The Discourse of Japanese Development Assistance and the Scaling-up of Community-based Health Planning and Services (CHPS) in Ghana | Kweku Ampiah | JICA Research Institute | 2015 | Commentary | - | - | Ghana | The paper discusses the contribution of Japan to CHPS in Ghana. |
| 41 | Effect of the National Health Insurance Scheme on Community-Based Health Planning and Services in the Vieri Zone of Wa West District of Ghana | Kanlisi Kaba Simon, Issaka Abdul-Hakim, Faustina Akwetey | Science Publishing Group | 2017 | Case Study | CHOs, the Community Health Management Committee (CHMC) and Community Health Volunteers | 165 households | Vieri, Gorziri and Lomwana in Upper West region | The main subject of the paper was to explore the effect of NHIS on CHPS |
| 42 | The Ghana Community-based Health Planning and Services Initiative for scaling up service delivery innovation | FRANK K NYONATOR, J KOKU AWOONOR-WILLIAMS, JAMES F PHILLIPS, TANYA C JONES3 AND ROBERT A MILLER | Oxford University Press, Health Policy and Planning | 2005 | Systematic review | CHOs, the Community Health Management Committee (CHMC) and Community Health Volunteers | - | Ghana | The main subject of the paper was to explore scaling Up of CHPS. |
| 43 | The role of community-based health planning and services strategy in involving males in the provision of family planning services: a qualitative study in Southern Ghana | Philip Baba Adongo, Placide Tapsoba, James F Phillips, Philip Teg-Nefaah Tabong |  | 2013 | Qualitative descriptive study | Community members, Health Workers, CHVs, CHOs, Public Health Nurses (PHNs),CHPS’ Coordinators at district and regional levels | 59 | Southern Ghana | The main subject of the paper was to explore male involvement in health care |
| 44 | Universal health coverage necessitates a system approach: an analysis of Community-based Health Planning and Services (CHPS) initiative in Ghana | Abraham Assan, Amirhossein Takian, Moses Aikins and Ali Akbarisari | BMC | 2018 | Qualitative design | Policy makers of the CHPS initiative, managers of CHPS compound and health centers, politicians, academics, health professionals, technocrats,and community health management committee members experts | 67 | Four regions of Ghana | The main subject of the paper was to explore CHPS and Universal Health Coverage to necessitate a system approach. |
| 45 | Uptake of Task-Strengthening Strategy for Hypertension (TASSH) Control within Community-Based Health Planning Services in Ghana: study protocol for a cluster randomized controlled trial | Kwaku Poku Asante, Juliet Iwelunmor, Kingsley Apusiga, Joyce Gyamfi4 | Biomed Central | 2020 | Hybrid clinical effectiveness implementation design | Adult uncontrolled Hypertensive patients | 700 | Bono East Region of Ghana. | The main subject of the paper was to explore task-Strengthening Strategy for Hypertension |
| 46 | Volunteer responsibilities, motivations and challenges in implementation of the community-based health planning and services (CHPS) initiative in Ghana: qualitative evidence from two systems learning districts of the CHPS+ project | Margaret Kweku, Emmanuel Manu, Hubert Amu, Fortress Yayra Aku, Martin Adjuik | BMC Health Services Research | 2020 | Focus Group Discussion (FGD) | Community Health Volunteers | 37 | Central Tongu District and Nkwanta South Municipality of Volta Region | The main subject of the paper was to explore community Volunteerism and contribution to CHPS and the responsibilities, motivations and challenges of community health management committees (CHMCs) in two CHPS+ Project districts in Ghana. |
| 47 | Willing but unable? Extending theory to investigate community capacity to participate in Ghana’s Community-based Health Planning and service implementation | Roger A. Atinga, Irene Akua Agyepong, Reuben K. | Evaluation and Program Planning | 2018 | Focus Group Discussion (FGD) | Traditional authorities, Assembly Members, Community Health Volunteers (CHV), and Community Health Management Committee Members (CHMC), FLP and district health managers (Directors and CHPS Coordinators) | 74 | Ghana | The main subject of the paper was to explore community capacity and to assess the level of community capacity to participate in the Community-Based Health Planning and Service (CHPS) in Ghana. |
| 48 | Utilization of community-based health planning and services compounds in the Kintampo North Municipality: a crosssectional descriptive correlational study | Kenneth Wiru, Akwasi Kumi-Kyereme, Emmanuel N. Mahama, Seeba Amenga-Etego | Bio Med Central | 2017 | descriptive cross-sectional correlational design f | Households in the Municipality | 171 households | Kintampo North Municipality in Brong Ahafo Region | The main subject of the paper was to explore utilization of Chps |
| 49 | The challenges and prospects of community-based health planning and services (CHPS) in the Nadowli district of the Upper West region, Ghana | Richmond Bambu Dombo | UDS Space | 2012 | Descriptive cross-sectional research design | - | - | Nadowli, Upper West Region | The main subject of the paper was to explore challenges of CHPS in Nadwoli. |
| 50 | The Community-based health planning and services (CHPS) and access to health care in the Ashanti region, Ghana | Victoria Yeleduor | UG Space | 2012 | Experimental design | Household heads | 80 | Ashanti region | The main subject of the paper was to explore access to CHPS in the Ashanti region. |
| 51 | A qualitative appraisal of stakeholders’ perspectives of a community-based primary health care program in rural Ghana | Mawuli K. Kushitor, Adriana A. Biney, Kalifa Wright | BMC | 2019 | qualitative(focus group discussion) | mothers and fathers with cihldren U5, commuinty leaders,and female adolescents without children | 126 | Volta and northern region | The main subject of the paper was to explore stakeholders appraisal |
| 52 | Evaluation of a community-based hypertension improvement program (ComHIP) in Ghana: data from a baseline survey | Peter Lamptey, Amos Laar, Alma J Adler | BMC PUBLIC HEALTH | 2017 | qualitative (cohort study) | adults 18+ | 2400 | Lower Manya Krobo district | The main subject of the paper was to explore hypertension improvement |
| 53 | Lessons learned from scaling up a community-based health program in the Upper East Region of northern Ghana | John Koku Awoonor-Williams, Elias Kavinah Sory, Frank K Nyonator, | Global health; science and practice | 2013 | Review of archivals reports | DHMT,RHMT reports | - | Upper east region | The main subject of the paper was to explore lessons from scaling up community-based program |
| 54 | Local patterns of social capital and sustenance of the Community-Based Health Planning Services (CHPS) policy: a qualitative comparative study in Ghana | Padmore Adusei Amoah | BMJ open | 2019 | qualitative (in-depth interviews) | young Adults | 32 | Apem,and Amo | The main subject of the paper was to explore the sustenance of CHPS. |
| 55 | Male involvement in maternal healthcare through Community- based Health Planning and Services: the views of the men in rural Ghana | Bassoumah Bougangue and How Kee Ling | BMC Public Health | 2017 | qualitative(focus group discussion and individual) | husbands | 93 | Awutu Senya west | The main subject of the paper was to explore male involvement in health care through CHPS |
| 56 | Retention and sustainability of community-based health volunteers' activities: A qualitative study in rural Northern Ghana | Samuel Chatio, Patricia Akweongo | PLoS ONE | 2017 | qualitative(indepth interviews) | community health volunteers | 32 | Kassena nankana east and west district | The main subject of the paper was to explore the sustainability of community-based volunteers |
| 57 | Using the community-based health planning and services program to promote skilled delivery in rural Ghana: socio-demographic factors that influence women utilization of skilled attendants at birth in northern Ghana | Evelyn Sakeah, Henry V Doctor, Lois McCloskey, | Reproductive health journal | 2014 | qualitative(intrinsic case study) | CHOs, district health directors, MCH units | 41 | Kassena nankana east, kassena nankana west and bongo district | The main subject of the paper was to explore the integration of skill birth attendance and community health officers in CHPS |
| 58 | Adapting the Community-based Health Planning and Services (CHPS) to engage poor urban communities in Ghana: protocol for a participatory action research study | Mary Abboah-Offei ,  Akosua Gyasi Darkwa,  Andrews Ayim, Adelaide Maria Ansah-Ofei,  Delanyo Dovlo,  John K Awoonor‐Williams, Erasmus Emmanuel Akurugu Agongo,  Irene Akua Agyepong,  Helen Elsey | BMJ | 2021 | Participatory Action Research | Urban residents, households vulnerable to ill-health and CHPS staff and key stakeholders | - | Old Fadama (Yam and Onion Market community), Adedenkpo and Adotrom | The main subject of the paper was to explore CHPS adaptation to engage poor urban communities. |
| 59 | A Collaborative Health Promotion Approach to Improve Rural Health Delivery and Health Outcomes in Ghana: A Case Example of a Community-Based Health Planning and Services (CHPS) Strategy | Awolu Adam, Adam Fusheini and Daniel Dramani Kipo-Sunyehzi | Intech Open | 2021 | Case study | - | - | Ghana | The main subject of the paper was to explore collaborative Health Promotion Approach to Improve Rural Health Delivery and Health Outcomes in Ghana |

**REFERENCES**

Abboah-Offei, M., Darkwa, A. G., Ayim, A., Ansah-Ofei, A. M., Dovlo, D., Awoonor‐Williams, J. K., ... & Elsey, H. (2021). Adapting the Community-based Health Planning and Services (CHPS) to engage poor urban communities in Ghana: protocol for a participatory action research study. *BMJ open*, *11*(7), e049564.

Adam, A., Fusheini, A., & Kipo-Sunyehzi, D. D. (2021). A Collaborative Health Promotion Approach to Improve Rural Health Delivery and Health Outcomes in Ghana: A Case Example of a Community-Based Health Planning and Services (CHPS) Strategy.

Adongo, P. B., Tapsoba, P., Phillips, J. F., Tabong, P. T. N., Stone, A., Kuffour, E., ... & Akweongo, P. (2013). The role of community-based health planning and services strategy in involving males in the provision of family planning services: a qualitative study in Southern Ghana. Reproductive health, 10(1), 1-15.

Aikins, A. D. G., Kushitor, M., Koram, K., Gyamfi, S., & Ogedegbe, G. (2014). Chronic non-communicable diseases and the challenge of universal health coverage: insights from community-based cardiovascular disease research in urban poor communities in Accra, Ghana. *BMC public health*, *14*(2), 1-9.

Amoah, P. A. (2019). Local patterns of social capital and sustenance of the Community-Based Health Planning Services (CHPS) policy: a qualitative comparative study in Ghana. *BMJ open*, *9*(2), e023376.

Ampiah, K. (2017). *The Discourse of Japanese Development Assistance and the Scaling-up of Community-based Health Planning and Services (CHPS) in Ghana* (No. 149). JICA Research Institute.

Ampiah, K. (2018). ‘Noso-politics’ and Japanese Development Assistance: The Scaling-up of Community-based Health Planning and Services (CHPS) in Ghana.

Apanga, P. A., & Adam, M. A. (2015). Factors influencing the uptake of family planning services in the Talensi District, Ghana. *Pan African Medical Journal*, *20*(1).

Asante, K. P., Iwelunmor, J., Apusiga, K., Gyamfi, J., Nyame, S., Adjei, K. G. A., ... & Plange-Rhule, J. (2020). Uptake of Task-Strengthening Strategy for Hypertension (TASSH) control within Community-Based Health Planning Services in Ghana: study protocol for a cluster randomized controlled trial. *Trials*, *21*(1), 1-13.

Assan, A., Takian, A., Aikins, M., & Akbarisari, A. (2019). Challenges to achieving universal health coverage through community-based health planning and services delivery approach: a qualitative study in Ghana. *BMJ open*, *9*(2), e024845.

Assan, A., Aikins, M., & Takian, A. (2019). Suicide in ghana: How could the community-based health planning and service (chps) effectively contribute to its prevention? *Iranian journal of public health*, *48*(11), 2097.

Atinga, R. A., Agyepong, I. A., & Esena, R. K. (2018). Ghana's community-based primary health care: why women and children are ‘disadvantaged’by its implementation. Social Science & Medicine, 201, 27-34.

Atuoye, K. N., Dixon, J., Rishworth, A., Galaa, S. Z., Boamah, S. A., & Luginaah, I. (2015). Can she make it? Transportation barriers to accessing maternal and child health care services in rural Ghana. *BMC health services research*, *15*(1), 1-10.

Awoonor-Williams, J. K., Bawah, A. A., Nyonator, F. K., Asuru, R., Oduro, A., Ofosu, A., & Phillips, J. F. (2013). The Ghana essential health interventions program: a plausibility trial of the impact of health systems strengthening on maternal & child survival. BMC health services research, 13(S2), S3.

Awoonor-Williams, J. K., Sory, E. K., Nyonator, F. K., Phillips, J. F., Wang, C., & Schmitt, M. L. (2013). Lessons learned from scaling up a community-based health program in the Upper East Region of northern Ghana. *Global Health: Science and Practice*, *1*(1), 117-133.

Awoonor Williams, J. K., Phillips, J. F., & Bawah, A. A. (2019). Scaling down to scale-up: a strategy for accelerating community-based health service coverage in Ghana. *Journal of Global Health Science*, *1*(1).

Baatiema, L., Skovdal, M., Rifkin, S., & Campbell, C. (2013). Assessing participation in a community-based health planning and services programme in Ghana. BMC health services research, 13(1), 233.

Bassoumah, B., Andani, M. A., & Adokiya, M. N. (2021). Challenges to the Utilization of Community-based Health Planning and Services: the views of stakeholders in Yendi Municipality, Ghana.

Bawah, A. A., Awoonor-Williams, J. K., Asuming, P. O., Boyer, C. B., Achana, S. F., Akazili, J., & Phillips, J. F. (2017, April). The child survival impact of the Ghana essential health interventions program: a health system strengthening initiative in a rural region of northern Ghana. In *Annual meeting of the population Association of America*.

Bawah, A. A., Kweku, M., Amu, H., Awolu, A., Adjuik, M., Ayanore, M. A., ... & Gyapong, J. O. (2020). Community-Based Health Planning and Services Plus programme in Ghana: A qualitative study with stakeholders in two Systems Learning Districts on improving the implementation of primary health care.

Begohn, M. M., Adei, D., & Mireku, I. S. (2016). Contribution of community-based health planning service in maternal health service delivery in the Tamale metropolitan area, Ghana. *J. Med. Physiol. Biophys*, *28*(0), 18-35.

Bougangue, B., & Ling, H. K. (2017). Male involvement in maternal healthcare through Community-based Health Planning and Services: the views of the men in rural Ghana. *BMC public health*, *17*(1), 1-10.

Chatio, S., & Akweongo, P. (2017). Retention and sustainability of community-based health volunteers' activities: A qualitative study in rural Northern Ghana. *PloS one*, *12*(3), e0174002.

Chatio, S., Welaga, P., Tabong, P. T. N., & Akweongo, P. (2019). Factors influencing performance of community-based health volunteers’ activities in the Kassena-Nankana Districts of Northern Ghana. *PloS one*, *14*(2), e0212166.

Dalaba, M. A., Stone, A. E., Krumholz, A. R., Oduro, A. R., Phillips, J. F., & Adongo, P. B. (2016). A qualitative analysis of the effect of a community-based primary health care programme on reproductive preferences and contraceptive use among the Kassena-Nankana of northern Ghana. *BMC Health Services Research*, *16*(1), 1-8.

Dalinjong, P. A., Welaga, P., Akazili, J., Kwarteng, A., Bangha, M., Oduro, A., ... & Goudge, J. (2017). The association between health insurance status and utilization of health services in rural Northern Ghana: evidence from the introduction of the National Health Insurance Scheme. *Journal of Health, Population and Nutrition*, *36*(1), 1-10.

de-Graft Aikins, A., Kushitor, M., Koram, K., Gyamfi, S., & Ogedegbe, G. (2014). Chronic non-communicable diseases and the challenge of universal health coverage: insights from community-based cardiovascular disease research in urban poor communities in Accra, Ghana. BMC public health, 14(2), 1-9.

Dil, Y., Strachan, D., Cairncross, S., Korkor, A. S., & Hill, Z. (2012). Motivations and challenges of community-based surveillance volunteers in the northern region of Ghana. *Journal of community health*, *37*(6), 1192-1198.

Dombo, R. B. (2012). *THE CHALLENGES AND PROSPECTS OF COMMUNITY-BASED HEALTH PLANNING AND SERVICES (CHPS) IN THE NADOWLI DISTRICT OF THE UPPER WEST REGION, GHANA* (Doctoral dissertation).

Esena, R. K. (2013). Managing for Quality in Health Care: Quality Improvement Issues in Ghana ‘s Community-based Health Planning and Services Concept. *Journal of Scientific & Innovative Research*, *2*(2).

Escribano Ferrer, B., Hansen, K. S., Gyapong, M., Bruce, J., Narh Bana, S. A., Narh, C. T., ... & Webster, J. (2017). Cost-effectiveness analysis of the national implementation of integrated community case management and community-based health planning and services in Ghana for the treatment of malaria, diarrhoea and pneumonia. *Malaria journal*, *16*(1), 1-18.

Ferrer, B. E., Webster, J., Bruce, J., Narh-Bana, S. A., Narh, C. T., Allotey, N. K., ... & Gyapong, M. (2016). Integrated community case management and community-based health planning and services: a cross sectional study on the effectiveness of the national implementation for the treatment of malaria, diarrhoea and pneumonia. *Malaria journal*, *15*, 1-15.

Haddaway, N. R., Page, M. J., Pritchard, C. C., & McGuinness, L. A. (2022). PRISMA2020: An R package and Shiny app for producing PRISMA 2020-compliant flow diagrams, with interactivity for optimised digital transparency and Open Synthesis Campbell Systematic Reviews, 18, e1230. <https://doi.org/10.1002/cl2.1230>

Johnson, F. A., Frempong-Ainguah, F., Matthews, Z., Harfoot, A. J., Nyarko, P., Baschieri, A., ... & Atkinson, P. M. (2015). Evaluating the impact of the community-based health planning and services initiative on uptake of skilled birth care in Ghana. *PLoS One*, *10*(3), e0120556.

Kanmiki, E. W., Akazili, J., Bawah, A. A., Phillips, J. F., Awoonor-Williams, J. K., Asuming, P. O., ... & Aikins, M. (2019). Cost of implementing a community-based primary health care strengthening program: The case of the Ghana Essential Health Interventions Program in northern Ghana. *PLoS One*, *14*(2), e0211956.

Krumholz, A. R., Stone, A. E., Dalaba, M. A., Phillips, J. F., & Adongo, P. B. (2015). Factors facilitating and constraining the scaling up of an evidence-based strategy of community-based primary care: management perspectives from northern Ghana. *Global public health, 10*(3), 366-378.

Kushitor, M. K., Biney, A. A., Wright, K., Phillips, J. F., Awoonor-Williams, J. K., & Bawah, A. A. (2019). A qualitative appraisal of stakeholders’ perspectives of a community-based primary health care program in rural Ghana. *BMC health services research*, *19*(1), 675.

Kweku M, Amu H, Awolu A, Adjuik M, Ayanore MA, et al. (2020) Community-Based Health Planning and Services Plus programme in Ghana: A qualitative study with stakeholders in two Systems Learning Districts on improving the implementation of primary health care. PLOS ONE 15(1): e0226808.

Kweku, M., Amu, H., Adjuik, M., Manu, E., Aku, F. Y., Tarkang, E. E., ... & Gyapong, J. O. (2020). Community involvement and perceptions of the community-based health planning and services (CHPS) strategy for improving health outcomes in Ghana: Quantitative comparative evidence from two system learning districts of the CHPS+ project. *Advances in Public Health*, *2020*.

Kweku, M., Amu, H., Adjuik, M., Aku, F. Y., Manu, E., Tarkang, E. E., ... & Gyapong, J. O. (2020). Community utilisation and satisfaction with the community-based health planning and services initiative in Ghana: a comparative study in two system learning districts of the CHPS+ project. *BMC health services research*, *20*(1), 1-14.

Kweku, M., Manu, E., Amu, H., Aku, F. Y., Adjuik, M., Tarkang, E. E., ... & Alornyo, J. S. (2020). Volunteer responsibilities, motivations and challenges in implementation of the community-based health planning and services (CHPS) initiative in Ghana: qualitative evidence from two systems learning districts of the CHPS+ project. *BMC Health Services Research*, *20*(1), 1-13.

Lamptey, P., Laar, A., Adler, A. J., Dirks, R., Caldwell, A., Prieto-Merino, D., ... & Perel, P. (2017). Evaluation of a community-based hypertension improvement program (ComHIP) in Ghana: data from a baseline survey. *BMC Public Health*, *17*(1), 1-16.

MacLeod, B., Phillips, J., Stone, A. E., Walji, A., & Awoonor-Williams, J. K. (2012). The architecture of a software system for supporting community-based primary health care with mobile technology: the mobile technology for community health (MoTeCH) initiative in Ghana. *Online Journal of Public Health Informatics*, *4*(1).

Naariyong, S., Poudel, K. C., Rahman, M., Yasuoka, J., Otsuka, K., & Jimba, M. (2012). Quality of antenatal care services in the Birim North District of Ghana: contribution of the community-based health planning and services program. *Maternal and child health journal*, *16*(8), 1709-1717.

Nartey, A. K., & Manful, E. SUSTAINING HEALTH INTERVENTION PROGRAMMES IN GHANA: THE ROLE OF THE ‘COMMUNITY’IN THE COMMUNITY-BASED HEALTH PLANNING AND SERVICES. *Money*, *3*, 1-88.

Ntsua, S., Tapsoba, P., Asare, G. Q., & Nyonator, F. K. (2012). Repositioning community-based family planning in Ghana: A case study of Community-based Health Planning and Services (CHPS).

Nyonator, F. K., Awoonor-Williams, J. K., Phillips, J. F., Jones, T. C., & Miller, R. A. (2003). The Ghana Community-based Health Planning and Services Initiative: fostering evidence-based organizational change and development in a resource-constrained setting.

Nyonator, F., Jones, T. C., Miller, R. A., Phillips, J. F., & Awoonor-Williams, J. K. (2003). Guiding the Ghana community-based health planning and services approach to scaling up with qualitative systems appraisal. *International Quarterly of Community Health Education*, *23*(3), 189-213.

Nyonator, F.K., Awoonor-Williams, J.K., Philips, J.F., & Jones, T.C (2005). The Ghana Community-based Health Planning and Services Initiative for scaling up service delivery innovation. *Health policy and planning*, 25-34

Phillips, J. F., Bawah, A. A., & Binka, F. N. (2006). Accelerating reproductive and child health programme impact with community-based services: the Navrongo experiment in Ghana. *Bulletin of the World Health Organization*, *84*, 949-955.

Sakeah, E., McCloskey, L., Bernstein, J., Yeboah-Antwi, K., Mills, S., & Doctor, H. V. (2014). Can community health officer-midwives effectively integrate skilled birth attendance in the community-based health planning and services program in rural Ghana? *Reproductive Health*, *11*(1), 1-13.

Sakeah, E., McCloskey, L., Bernstein, J., Yeboah-Antwi, K., Mills, S., & Doctor, H. V. (2014). Is there any role for community involvement in the community-based health planning and services skilled delivery program in rural Ghana? *BMC health services research*, *14*(1), 1-14.

Sakeah, E., Doctor, H. V., McCloskey, L., Bernstein, J., Yeboah-Antwi, K., & Mills, S. (2014). Using the community-based health planning and services program to promote skilled delivery in rural Ghana: socio-demographic factors that influence women utilization of skilled attendants at birth in Northern Ghana. *BMC public health*, *14*(1), 1-9.

Sakeah, E., Aborigo, R., Sakeah, J. K., Dalaba, M., Kanyomse, E., Azongo, D., ... & Oduro, A. R. (2018). The role of community-based health services in influencing postnatal care visits in the Builsa and the West Mamprusi districts in rural Ghana. *BMC pregnancy and childbirth*, *18*(1), 1-9.

Simon, K. K., Issaka, A. H., & Akwetey, F. (2017). Effect of the national health insurance scheme on community-based health planning and services in the Vieri zone of Wa West district of Ghana.

Smith, K. V., & Sulzbach, S. (2008). Community-based health insurance and access to maternal health services: evidence from three West African countries. *Social science & medicine*, *66*(12), 2460-2473.

Vaismoradi, M., Jones, J., Turunen, H., & Snelgrove, S. (2016). Theme development in qualitative content analysis and thematic analysis. 6(5). <https://doi.org/10.5430/jnep.v6n5p100>

Wiru, K., Kumi-Kyereme, A., Mahama, E. N., Amenga-Etego, S., & Owusu-Agyei, S. (2017). Utilization of community-based health planning and services compounds in the Kintampo North Municipality: a cross-sectional descriptive correlational study. BMC health services research, 17(1), 679.

Woods, H. M. (2016). The Contribution of Community-based Health Planning and Services (CHPS) to Community Sustainability and Health in the Upper West Region of Ghana.

Woods, H., & Luginaah, I. (2018). The influence of the Community ‐ based Health Planning and Services (CHPS) program on community health sustainability in the Upper West Region of Ghana. (August), 1–15. https://doi.org/10.1002/hpm.2694

Wright, K. J., Biney, A., Kushitor, M., Awoonor-Williams, J. K., Bawah, A. A., & Phillips, J. F. (2020). Community perceptions of universal health coverage in eight districts of the Northern and Volta regions of Ghana. *Global Health Action*, *13*(1), 1705460.

Yeleduor, V. (2012). *The community-based health planning and services (CHPS) and access to health care in the Ashanti Region, Ghana* (Doctoral dissertation).
